# Reactive Astrocytes as Therapeutic Targets for Brain Degenerative Diseases: Roles Played by Metabotropic Glutamate Receptors

**DOI:** 10.1007/s11064-020-02968-6

**Published:** 2020-01-25

**Authors:** Talia M. Planas-Fontánez, Cheryl F. Dreyfus, Kyle S. Saitta

**Affiliations:** 1 Joint Graduate Program in Toxicology, Rutgers, The State University of New Jersey, Piscataway, NJ USA; 2 Department of Neuroscience and Cell Biology, Rutgers Robert Wood Johnson Medical School, Piscataway, NJ USA; 3 Robert Wood Johnson Medical School, 683 Hoes Lane West, Room 361, Piscataway, NJ 08854 USA

**Keywords:** Astrocyte heterogeneity, Group I/II metabotropic glutamate receptors, Multiple sclerosis, Alzheimer’s disease, Amyotrophic lateral sclerosis

## Abstract

Astrocytes are well known to play critical roles in the development and maintenance of the central nervous system (CNS). Moreover, recent reports indicate that these cells are heterogeneous with respect to the molecules they express and the functions they exhibit in the quiescent or activated state. Because astrocytes also contribute to pathology, promising new results raise the possibility of manipulating specific astroglial populations for therapeutic roles. In this mini-review, we highlight the function of metabotropic glutamate receptors (mGluRs), in particular mGluR3 and mGluR5, in reactive astrocytes and relate these to three degenerative CNS diseases: multiple sclerosis, Alzheimer’s disease and Amyotrophic Lateral Sclerosis. Previous studies demonstrate that effects of these receptors may be beneficial, but this varies depending on the subtype of receptor, the state of the astrocytes, and the specific disease to which they are exposed. Elucidating the role of mGluRs on astrocytes at specific times during development and disease will provide novel insights in understanding how to best use these to serve as therapeutic targets.

## Introduction

**Fig. 1:**
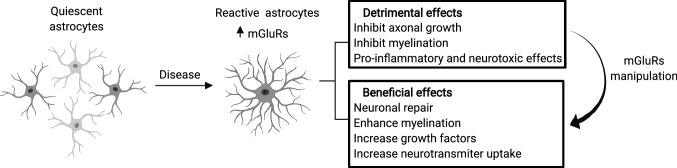
Response of astrocytes to disease can be manipulated by mGluRs in a positive or negative direction depending on the state of astrocyte activation, the local astrocyte environment and the disease that is responsible for reactivity. Created with BioRender

It is well recognized that astrocytes play a number of critical roles that support the developing and mature brain. In response to injury, however, astrocytes exhibit profound changes in these roles that can result in both negative and positive influences on surrounding cells. We suggest that these roles can be harnessed to aid in the recovery from injury. In particular, we focus this mini-review on roles played by metabotropic glutamate receptors (mGluRs) that are expressed on astrocytes during disease. Recent studies suggest that stimulation of these receptors in some cases may elicit protective effects on neighboring cells and may represent a new therapeutic approach to brain dysfunction. In other cases, however, antagonism may be preferable. Therefore, caution is warranted when evaluating effectiveness of mGluR stimulation (see Fig. 1). As indicated in this mini-review, astrocytes whether quiescent or reactive are highly heterogeneous populations with respect to their response to the local central nervous system (CNS) region in which they reside, and the specific diseases or injuries to which they are exposed. Therefore, the utility of application of specific agonists or antagonists may vary depending on the specific astrocytic populations under investigation and how they are impacted by their environment.

Before beginning it should be noted that we limit this review to astrocytes, their response to injury and effects of mGluR agonists. We recognize that other glial cells react to mGluR stimulation as well as other activating influences. For a more comprehensive analysis of responses of these cells we refer the reader to excellent additional reviews of this subject [1–5].

## Astrocytic Function in the Unlesioned brain

Astrocytes are the most abundant glial cell in the CNS and are specialized to perform many functions to support neuronal activity in the developing and adult nervous system. These include ion homeostasis, uptake of neurotransmitters, release of growth factors, participation in synaptic transmission, regulation of the blood-brain barrier and contribution to the CNS immune system [6]. Astrocytes also present a dynamic environment for axon guidance during development by providing appropriate cell surface receptors and adherent molecules [7].

Interestingly, many of the functions of astrocytes are regulated by neuron-to-astrocyte crosstalk. Astrocytes are able to respond to several neurotransmitters, including glutamate [8, 9], adenine triphosphate (ATP) [10, 11], gamma-aminobutyric acid (GABA) [12, 13], acetylcholine [14] and endocannabinoids [15]. In response to these transmitters, astrocytes elevate intracellular calcium levels, release a number of gliotransmitters as well as a host of growth factors that impact neuronal function [16, 17]. As a result of such signaling, astrocytes then modulate synaptic function, maintenance, pruning and remodeling and express ion channels and neurotransmitter receptors and transporters [15, 18, 19]. These physiological roles, manipulated by gliotransmitters and growth factors in normal astrocytes, are also observed during pathophysiological states of the nervous system, as discussed below.

Astrocytes are heterogeneous cells that not only differ in morphology and expression of intermediate filament levels, but also in the roles they play [20]. For example, morphological differences are reported when a subtype of astrocytes from the human cortex and hippocampus are compared. High levels of glutamine synthetase (GS) and excitatory amino-acid transporters − 1 and − 2 (EAAT1, EAAT2) are observed in the hippocampus with long-process astrocytes, while cortical astrocytes are more heterogeneous with cells that are protoplasmic but exhibit reduced numbers of small processes and a low expression of GS, EAAT1, and EAAT2 [21]. As suggested here, the heterogeneity in morphology may extend to heterogeneity in function. This is indicated for example in the observation that astrocytes of different regions release different substances that may influence neighboring neurons distinctly. For example, cultured astrocytes of the substantia nigra are better at supporting dopamine neuron survival than are astrocytes of the hippocampus [22]. Such differences are also noted when substantia nigra astrocytes are compared to those of the ventral tegmental area. In this case, recent studies of effects of astrocytes on local dopaminergic neurons suggest that growth and differentiation factor 15 (GDF15), a member of the transforming growth factor beta (TGF-β) superfamily, may be responsible for differences in survival and protection when dopaminergic neurons from the two brain regions are compared [23].

Heterogeneity also is observed in the markers of astrocyte function within brain regions. For example, astrocytic α-amino-3-hydroxy-5-methyl-4-isoxazolepropionic acid (AMPA) receptors, glutamate transporter 1, and potassium channel Kir4.1 expression are differentially expressed within regions as well as between specific regions in the brain [24–27]. With respect to expression of mGluR5, examined in this mini-review, heterogeneity is also evident. Thus, few astrocytes of the spinal cord exhibit this receptor [28], while the majority of cortical astrocytes do [29]. It is important to consider what roles these differences may play when thinking of how regional astrocytes may differ in response to mGluR agonists in distinct disease states.

## Astrocytic Response to Injury

In the case of brain injury or disease, astrocytes become reactive. In this process, many of the actions of quiescent cells become enhanced or reduced to influence proximate cells. Traditionally, it was thought that these changes are negative. For example, astrocytes can form a physical barrier to axon growth and produce a variety of molecules that serve as an impediment to nerve cell survival [30, 31]. Moreover, reactive astrocytes express a wide variety of inflammation-associated molecules and are capable of antigen presentation. These changes have profound pro-inflammatory effects that present an inhibitory environment for glial differentiation and endogenous remyelination [32, 33]. In a more specific example, studies by the Gallo laboratory demonstrated that when effects of endothelin-1, a secreted intercellular signaling molecule were characterized after focal demyelination of the corpus callosum, it acted as a negative regulator of NG2 glial differentiation and functional remyelination [32]. Moreover, ablation or inhibition of endothelin receptor-B accelerates oligodendrocyte progenitor differentiation and remyelination [33]. Similarly, other proteins, such as bone morphogenetic proteins, have negative effects on oligodendrocytes following spinal cord injury (SCI) [34] and in the case of the mouse model of ALS, mutated astrocytes can release toxic factors that kill up to 90% of co-cultured motor neurons [35, 36]. This has relevance to the response to disease states.

On the other hand, recently, it has become more widely recognized that astrocytes can also have neuroprotective effects and enhance axonal and neuronal regeneration [37, 38]. Reactive astrocytes in some cases suppress immune responses following CNS injury, maintain extracellular homeostasis and produce growth factors [39]. Thus, newly proliferated astrocytes may interact and organize into scars that surround and isolate tissue lesions and protect or enhance regeneration. For example, after SCI [40], signal transducer and activator of transcription 3 (STAT3), expressed by reactive astrocytes, has a key role in regeneration that includes control of inflammation [41, 42]. Selective deletion of the Stat3 driver after a wound leads to a significant increase of immune cell infiltration and neurodegeneration [38, 43]. These observations suggest that astrocytes may be critical for the recovery of function and survival after injury.

To evaluate the effects of injury on astrocyte function and their production of specific molecules, gene transcriptome approaches have been used to characterize subtypes of astroglial cells in response to brain damage. GeneChip analysis of reactive astrocytic populations was evaluated in two brain injury mice models: neuroinflammation induced by a single intraperitoneal injection of lipopolysaccharide and focal ischemic stroke produced by transient middle cerebral artery occlusion [44]. In both models, glial fibrillary acidic protein immunoreactivity is observed after 1 day and persists at least 1 week in combination with increased activated microglia. A core set of genes is upregulated in reactive astrocytes of both injury models, however, at least 50% of the altered gene expression is specific to a given injury type. These data suggest that there are distinct subtypes of reactive astrocytes, reminiscent of distinct types of quiescent astrocytes. In the case of reactive astrocytes, these have been termed as A1 and A2 based on their detrimental or beneficial effects, respectively, during injury and repair [44–46].

A1 reactive astrocytes may have negative effects on surrounding cells in response to inflammation. For example, they may secrete molecules that are inhibitory to neurite outgrowth. In addition, swelling of these astrocytes after injury may result in the release of excessive amounts of glutamate. Liddelow et al. [46] suggests that this A1 activation may be induced by activated microglia through the secretion of cytokines. After induction, A1 astrocytes secrete a neurotoxin of uncertain identity that induces rapid death of neurons and oligodendrocytes. A2 astrocytes, in contrast, are commonly induced by ischemia and their responses to the ischemia are beneficial. This population is geared toward restoring trophic support and synapse repair and to promote the survival and growth of neurons [47]. A2 astrocytes express high levels of neurotrophic factors and cytokines, including brain-derived neurotrophic factor (BDNF), cardiotrophin-like cytokine factor 1 (CLCF1), interlukin-6 (IL-6), and GDF15, as well as thrombospondins that promote synapse repair [44, 48]. Determining the cellular and molecular basis underlying A2 induction remains an issue to address and is important with respect to degenerative disease.

## Use of mGluRs to Regulate Astrocytes After Injury

A number of studies have focused on astrocytic mGluRs as targets that can be manipulated to enhance repair after injury [5, 31, 45, 49, 50]. mGluRs, particularly mGluR3 and mGluR5, are the two most abundant mGluRs found on astrocytes [29, 51]. In response to injury, these receptors are upregulated at the lesion site, suggesting that astrocyte function can be influenced in the specific location where effects may be important. However, as noted previously, discretion is merited with respect to the function of these receptors. While some studies have described positive effects of astrocytic mGluR activation after injury through the actions of neurotrophins and growth factors [50, 52–55], others have reported that they may elicit harmful effects through the production of cytokines and inflammatory mediators [56]. These differences in effect may be due to the state of activation of astrocytes, the region being assessed and the type of lesion being examined [29, 45, 46, 57, 58]. Consequently, environmental distinctions must be taken into consideration when assessing astrocytic mGluRs as potential pharmacological targets and included in determining whether stimulation of these receptors should be enhanced or inhibited.

## Signaling by and Regional Expression of Astrocytic mGluR3 and mGluR5

mGluRs are G protein-coupled receptors consisting of seven transmembrane domains that are subdivided into Group I, II, and III based on their signaling transduction pathways, amino acid sequence homology, and selectivity of agonists and antagonists [1, 2, 5, 59, 60]. mGluR5, in addition to mGluR1, is classified as a Group I mGluR, while mGluR3 is part of the Group II mGluRs along with mGluR2. Group I mGluRs function through G_q_-proteins, resulting in activation of phospholipase C (PLC), hydrolysis of phosphoinositides, release of calcium, and activation of protein kinase C (PKC). Further downstream signaling pathways include casein kinase 1, cyclin-dependent protein kinase 5, Jun kinase, mitogen-activated protein kinase/extracellular receptor kinase, and mammalian target of rapamycin/p70 S6 kinase [61–66]. On the other hand, Group II mGluRs are associated with G_i_- and G_0_-proteins, and are negatively coupled to adenylate cyclase. Activation of Group II mGluRs inhibits voltage-gated calcium entry into the cell. In addition, these receptors can activate MAPK and phosphatidyl inositol 3-kinase pathways [1, 2, 5, 59, 67, 68].

Gene expression analysis has been done to study the presence of mGluR3 and mGluR5 specifically on astrocytes isolated from mouse hippocampus or cortex. These studies reveal that while all mGluRs are found at least at low levels in adult tissue in mice, the most abundant receptor is mGluR3 followed by mGluR5 [51]. These two mGluRs are also present in humans under normal conditions, while others are undetectable [51, 69–71]. It is of interest that in some cases expression of the receptors changes with development, but not in others, suggesting that the role of specific receptors may be altered as the brain matures. Astrocytic mGluR3 expression remains relatively stable at 1-, 2-, 3-, and 12-weeks of age [51], while mGluR5 expression is highest at postnatal day 7 [51, 72] before rapidly declining through adulthood [51, 72].

To examine the presence of these receptors in astrocytes in vitro, cells are removed from developing animals and grown in culture. This approach has the advantage of evaluating the isolated cells, examining their receptors and defining their function. In general, as was the case in vivo, mGluR3 and mGluR5 show strong expression when compared to all the other mGluRs [29]. Interestingly, culture studies also reveal that regional differences are apparent. While mGluR3 and mGluR5 are found in astrocytes isolated from thalamus, tegmentum, cortex, hippocampus, and striatum [29], there are almost undetectable levels of these receptors within the cerebellum [29] and spinal cord [28]. It is interesting to consider what these regional differences may signify. Transcriptome analysis has indicated that cultured astrocytes exhibit a phenotype akin to A2 reactive astrocytes of the ischemic brain [44]. These studies suggest that regional differences in astrocyte expression of mGluRs in culture may foretell regional differences that while not evident in vivo, will be evident after specific injuries.

Rodent brain slices have been studied to bridge the gap between in vitro and in vivo studies. In particular, specific agonists of Group I and/or Group II mGluRs induce transient increases in intracellular calcium levels within astrocytes of hippocampal slices as they do in vivo [73–77]. In concordance with in vivo studies also is the fact that astrocytic mGluR5 is developmentally regulated in slices with the highest expression occurring in slices isolated from P1-10 rodents before declining into adulthood [78, 79].

In models of disease and in human disease tissue, levels of astrocytic mGluRs are upregulated in or near lesions. Therefore, we propose that the roles of these receptors may be most apparent during development, become downregulated during adulthood, but emerge to play critical roles during CNS disease. The models in which mGluR5 is elevated include multiple sclerosis (MS) [50], Alzheimer’s disease (AD) [80], amyotrophic lateral sclerosis (ALS) [81], epilepsy [82–85] and SCI [86–88]. Similarly, astrocytic mGluR5 is upregulated in human tissue from patients with MS [69, 71], AD [89, 90], ALS [91, 92] and epilepsy [93–95]. In regards to mGluR3, its expression is enhanced on astrocytes in animal models of epilepsy [82, 83] and in human tissue taken from patients with MS [69, 71], ALS [91, 92] and epilepsy [94].

## Roles of mGluRs on Reactive Astrocytes

In general, stimulation of Group I and/or Group II mGluRs on reactive astrocytes leads to the release of neurotransmitters, including glutamate [29, 73, 96, 97], as well as other factors such as BDNF [55, 98], glial-derived neurotrophic factor (GDNF) [54], and TGF-β [52, 53]. Astrocytic mGluR activation can also lead to enhanced glutamate uptake through Group I or II receptors [99, 100]. These data suggest that astrocytic mGluRs have the potential to play positive roles in the diseased brain. Nevertheless, these effects may vary based on the different environments of the different diseases. For example, as will be discussed in the next section, mGluR stimulation may elicit positive astrocytic effects in diseases such as MS and AD, while eliciting mixed effects in other diseases like ALS.

## Roles of mGluR3 and mGluR5 in Response to Disease

### Multiple Sclerosis

mGluR5 is increased in reactive astrocytes specifically within the lesion sites of the cuprizone model of MS [50]. This increase is not found on microglia or CC1 + mature oligodendrocytes. In the experimental autoimmune encephalomyelitis (EAE) model of MS, studies of tissue samples taken from EAE rodents indicate an increase in mGluR5 in the whole brain and forebrain. However, the cells expressing these receptors were not identified [101, 102]. In an attempt to determine roles of mGluR agonists and antagonists in rodent models of MS, these drugs have been injected either locally within the lesion site or systemically. The Group I/Group II mGluR agonist *trans*-(1S,3R)-1-amino-1,3-cyclopentanedicarboxylic acid (ACPD) injected directly into the cuprizone-induced lesion increases synthesis and release of BDNF, an effect that is blocked when BDNF was selectively deleted from astrocytes, suggesting that the mGluRs mediate the increase in this trophic factor in astrocytes [50].

In the case of the EAE models, effects of the mGluR agonists and antagonists were injected into the whole animal making the relative contribution of these receptors on astrocytes compared to other cell types unknown. In this model, mGluR5 antagonists have no effect on motor function [103], nor do they affect myelin ultrastructure compared to EAE animals receiving vehicle [102, 104, 105], suggesting that actions of mGluRs in EAE may be different from those in the cuprizone model. It should be noted however, that application of the agonists to the EAE CNS as a whole may miss a subtle difference elicited through the actions of astrocytes that can be enhanced. Clearly, additional studies are needed to identify which cells express mGluRs. This makes it possible to elucidate roles of agonists and determine their potential to signal though mGluRs on astrocytes or other cell types.

### Alzheimer’s Disease

In vivo studies of mGluRs and astrocytes in AD are quite limited, however it is interesting to note that amyloid-beta (Aβ) increases expression of mGluR5 in an AD transgenic model [80]. This effect also occurs when Aβ is added to astrocytes in culture [80, 89, 90, 106], indicating that when this agent is elevated, roles of astrocytic mGluR5 may be enhanced. However, information is lacking as to what the consequence is of this upregulation.

Culture models of AD are most informative in defining effects of mGluRs on astrocytes. These indicate that mGluRs are present on astrocytes in these models. In general, stimulation of the receptors has had beneficial results. This is most well known with respect to Group II receptors. For example, astrocytic Group II activation reduces Aβ production [107], and increases Aβ uptake in astrocytes, as well as releases BDNF from these cells [55]. BDNF in this study enhances neuron survival when neurons are challenged by treatment with Aβ. In complementary work, stimulation with ACPD that stimulates both Group I and Group II receptors also increases BDNF synthesis and release [98]. Other studies indicate that stimulation of mGluR3 reduces Aβ-induced neurodegeneration in mixed neuronal-glia cultures [53]. This effect is blocked when the receptor function is inhibited or when astrocytes are derived from mGluR3 deleted mice. In this case, mGluR3 rescues the neurons from Aβ-elicited death through the action of astrocyte-derived TGF-β. Overall, these studies in culture models of AD indicate that both astrocyte-derived BDNF and TGF-β may play positive protective roles in this disease and that this may be regulated by mGluRs.

### Amyotrophic Lateral Sclerosis

In ALS, studies of the role of astrocytic mGluR5 have focused on astrocytes cultured from animal models of the disease, particularly the hSOD1^G93A^ mouse or rat models. These studies find that mGluR5 is expressed at three-fold greater levels in hSOD1^G93A^ astrocytes than in wild-type cells [81]. In this disease however, upregulation of astrocytic mGluR5 appears to have negative consequences. Stimulation of Group I mGluRs on hSOD1^G93A^ astrocytes results in the death of these cells and this effect is blocked with an mGluR5 antagonist [108]. Moreover, while wild-type astrocytes treated with a Group I agonist enhances aspartate uptake, astrocytes derived from hSOD1^G93A^ rats fail to increase aspartate uptake, indicating that the mutant gene blocks protective roles of the Group I agonist [81, 109]. Proper removal of excitatory transmitters such as aspartate and glutamate can be important in preventing excitotoxicity in diseases such as ALS, where increased glutamate levels and reduced glutamate transporter expression is evident in tissue from ALS patients [110, 111]. Inhibiting mGluR5 activity on hSOD1^G93A^ astrocytes is then a strategy that may be pursued to enhance protective astrocytic functions.

In contrast to activation of Group I receptors, potential actions of Group II mGluRs on hSOD1^G93A^ astrocytes have not yet been studied in culture. However, effects of a Group II agonist injected subcutaneously have been studied in hSOD1^G93A^ mice [54]. Injection results in reduced neuronal death and elevated GDNF levels in the spinal cord with corresponding improvements in motor performance and neurologic signs. The same study shows that the Group II agonist enhances GDNF release from cultured wild-type astrocytes through mGluR3. It is not yet known if astrocytes are responsible for the effects observed in hSOD1^G93A^ mice.

## Conclusions

This mini-review has documented a number of studies that suggest the possible importance of astrocytes as therapeutic targets in treatment of CNS disease. In particular, we summarize roles of quiescent astrocytes and how they alter their functions in response to injury. In discussing these events it becomes obvious that astrocytes are not simple homogeneous populations. Their critical impact on the maintenance of CNS function has been increasingly recognized. However, what is still generally unappreciated is their heterogeneity in function and in response to disease. We focus this review on the roles played by mGluR3 and mGluR5, recognizing that these receptors are only representative of multiple receptors that influence function. What has been obvious, however, is that these receptors are upregulated on astrocytes at or near lesion sites, putting them in optimal position to have important influences under these conditions. Moreover, manipulation of signaling through these receptors is beginning to emerge as a strategy worth pursuing in at least some disease conditions.

One note about the studies that have been discussed: Although descriptive work assesses how astrocytes respond to injury and where mGluRs are expressed on astrocytes, it has been difficult to attribute the results of manipulation of these cells and these receptors to function in vivo. Critical work is clearly necessary to extend studies of function by using new animal models where astrocytes specifically can be manipulated by the deletion of a particular protein at distinct time points as is now being done in a number of studies [50, 100].
